# Molecular detection using aptamer-modified gold nanoparticles with an immobilized DNA brush for the prevention of non-specific aggregation†

**DOI:** 10.1039/d0ra05149g

**Published:** 2021-03-24

**Authors:** Yuki Yano-Ozawa, Nadine Lobsiger, Yu Muto, Takahiro Mori, Ken Yoshimura, Yuki Yano, Wendelin Jan Stark, Mizuo Maeda, Tsuyoshi Asahi, Atsushi Ogawa, Tamotsu Zako

**Affiliations:** Department of Chemistry and Biology, Graduate School of Science and Engineering, Ehime University 2-5 Bunkyo Matsuyama Ehime 790-8577 Japan zako.tamotsu.us@ehime-u.ac.jp; Institute for Chemical and Bioengineering, ETH Zürich Wolfgang-Pauli-Strasse 10 CH-8093 Zürich Switzerland; Tokyo Research Center, TOSOH Corporation 2743-1 Hayakawa Ayase Kanagawa 252-1123 Japan; Bioengineering Laboratory, RIKEN Cluster for Pioneering Research 2-1 Hirosawa Wako Saitama 351-0198 Japan; Proteo-Science Center, Ehime University 3 Bunkyo Matsuyama Ehime 790-8577 Japan

## Abstract

Gold nanoparticles (AuNPs) are often used for biosensing. In particular, aptamer-modified AuNPs are often used for colorimetric molecular detection, where target molecule-induced AuNP aggregates can be recognized by a color change from red to blue. However, non-specific aggregation could be induced by various compounds, leading to false-positive results. In this work we employed high-density ssDNA modification on the AuNP surface to prevent non-specific aggregation. The covalently immobilized DNA brush was used as an anchor for an aptamer specific for the target molecule. Herein, as a proof-of-concept study, we demonstrated detection of estradiol (E2), one of the endocrine-disrupting estrogen molecules as a model target, in the presence of antibiotic kanamycin (KN) as a model of co-contaminating compounds that induce non-specific aggregation of AuNPs. We also developed a smartphone dark field microscope (DFM) to visualize AuNP aggregation. Our previous study demonstrated that the observation of light scattering by AuNP aggregates with DFM can be applied for versatile molecular detection. In this work, we could successfully detect E2 with the smartphone DFM, and the results were verified by the results from a conventional benchtop DFM. This study would contribute to the future field applicability of AuNP-based sensors.

## Abstract 2

We demonstrated molecular detection using aptamer-modified gold nanoparticles with DNA-brush for the prevention of non-specific aggregation and smartphone darkfield microscopy.

## Graphical abstract

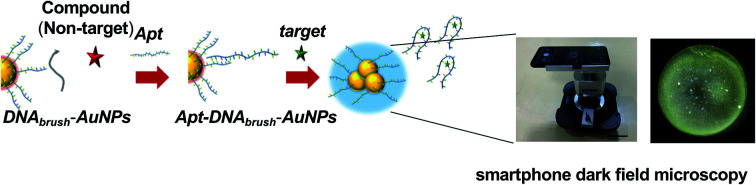

## Introduction

The distinct and exploitable chemical and physical properties (stability, activity, conductivity and surface chemistry) of gold nanoparticles (AuNPs) have led to a vast body of research describing their use for various applications.^1–5^ For example, the formation of AuNP aggregates can be recognized by a color change from red to blue/purple due to interparticle plasmon coupling, which leads to colorimetric biosensing of molecules that trigger aggregation of AuNPs, such as DNA, proteins, metal ions and chemical compounds.^6–11^

Aptamer-modified AuNPs are often used for colorimetric detection. Aptamers are short single-stranded oligonucleotides, which have been artificially selected *in vitro* and exhibit high affinity and specificity toward their respective ligands.^12–15^ While aptamer-modified AuNPs are stable even under high-salt conditions because of electric repulsion, the aptamer adsorbed on AuNP surface desorbs from the NP surface upon the addition of target molecules. Accordingly, the amount of the aptamer on the surface decreases, resulting in an unstable AuNP dispersion in the presence of salt, leading to the aggregation of the AuNPs. Zhao *et al.* have presented a structure-switching, aptamer-based, salt-induced AuNP sensor for the detection of adenosine.^16^ Wu *et al.* have described an AuNP–aptamer–surfactant biosensor for the detection of arsenic(iii) in aqueous solutions.^17^ However, non-specific aggregation could be induced by various compounds. For example, small molecules such as antibiotic kanamycin (KN) and amino acids could induce non-specific aggregation of AuNPs due to their positive charge and high partition coefficient values.^18,19^ The presence of such molecules could thereby lead to false-positive results.

**Fig. 1:**
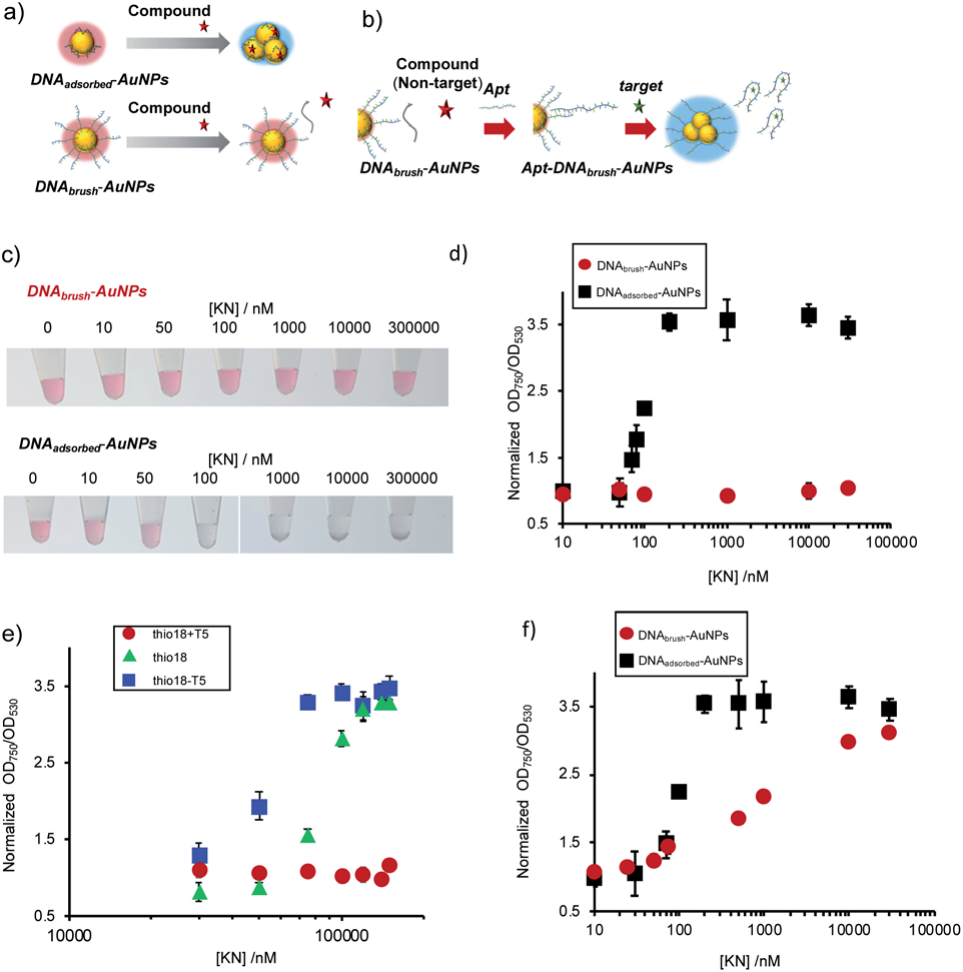
Inhibition of non-specific AuNP aggregation by dense thiol DNA modification. (a) Schematic of the aggregation of AuNPs when DNA was immobilized on the surface by physical adsorption (DNA_adsorbed_-AuNPs). AuNP surface modification with a DNA brush (Au–S bonds) (DNA_brush_-AuNPs) to inhibit non-specific aggregation by compounds such as KN. (b) Schematic of the surface modification with an aptamer hybridized to the immobilized DNA brush for the detection of a target substance. (c) Typical photos of the AuNPs at increasing KN concentrations (between 0–30 000 nM) showing aggregation when the DNA was only immobilized by physical adsorption (DNA_adsorbed_-AuNPs), whereas no aggregation was formed for DNA_brush_-AuNP. (d) Normalized OD_750_/OD_530_ values of the AuNP solutions. OD ratio without KN was defined as 1. The averaged values of three different samples tubes were shown. (e) Effect of KN on DNA_brush_-AuNP with different lengths (thio18 − T5 (13 bases), thio18 (18 bases), thio18 + T5 (23 bases)). The same amount of DNA (approximately 1100 molecules) was immobilized on the AuNP surface. The averaged values of three different samples tubes were shown. (f) Effect of KN on DNA_brush_-AuNP and DNA_adsorbed_-AuNP with the same amount of DNA (approximately 100 molecules). The averaged values of three different samples tubes were shown.

In this study, we employed high-density ssDNA modification on the AuNP surface to prevent non-specific aggregation. Previous reports showed that approximately 500–1000 DNA molecules could be immobilized on the AuNP surface (40 nm diameter) by using thiol-modified ssDNA and the thiol–Au interaction.^8,20,21^ We hypothesized that densely and covalently immobilized ssDNA could prevent non-specific aggregation by various compounds (Fig. 1a). We also conducted molecular detection using aptamer hybridized on high-density DNA modification on the AuNP surface in the presence of contaminating compounds. The hybridized aptamer will be released from the AuNP surface upon binding of target molecules. Differences in the surface charge will cause target-dependent AuNP aggregation (Fig. 1b).

Herein, as a proof-of-concept study, we investigated the detection of 17β-estradiol (E2) in the presence of KN as a model environmental water contaminant. E2 is one of endocrine-disrupting estrogen compounds, and was selected as a model target because of its frequent occurrence and its effects on the environment and human health.^22,23^ Although the detection of E2 using AuNPs and DNA aptamers has been reported,^24–26^ non-specific aggregation of AuNPs by possible contaminants has not been considered. KN was also selected as a model contaminant since KN has previously been shown to be the antibiotic showing the strongest non-specific aggregating effect on AuNPs.^19^ In this work, the potential of AuNPs modified with densely immobilized DNA for the suppression of non-specific aggregation in combination with a DNA aptamer specific for E2 was demonstrated.

Additionally, we built a smartphone dark field microscope (DFM) to visualize AuNP aggregation and to contribute to the future field applicability of AuNP-based sensors. Our previous study demonstrated that the observation of light scattering by AuNP aggregates with DFM can be applied for versatile molecular detection.^27^ Sun *et al.* also demonstrated that a smartphone DFM could be used to observe AuNPs for protein quantification assays.^28^ In the work at hand, we could successfully detect E2 with the smartphone DFM by observing AuNP aggregates, and the results were verified by the results by a conventional benchtop DFM. The use of smartphones leads to easy to handle, cost-effective and portable novel analytical solutions. By engineering AuNP surfaces with increased specificity, the versatility of the AuNP–aptamer detection system can be expanded to the detection of other targets.

## Experimental

### Materials

AuNPs (40 nm) were obtained from BBI solutions (Cardiff, UK). The E2 aptamer (E2 aptamer 47, 5′-CTCTCGGGACGACATGGATTTTCCATCAACGAAGTGCGTCCGTCCCG-3′),^29^ aptamer sequence-scrambled DNA (random DNA) (5′-CTCTCGGGACGACTTCAATGCACTTGGCGGCGACTACATCTCGGCAT-3′) and non-thiolated DNA (5′-GTCGTCCCGAGAGTTTTT-3′) were purchased from Eurofins Genomics (Tokyo, Japan). Thiolated DNAs (thio18, 5′-GTCGTCCCGAGAGTTTTT-SH-3′, thio18 + T5, 5′-GTCGTCCCGAGAGTTTTTTTTTT-SH-3′, thio18 − T5, 5′-GTCGTCCCGAGAG-SH-3′) were purchased from Invitrogen (Carlsbad, USA). E2, estrone (E1), estriol (E3), deoxycholic acid (DCA), cholesterol (Chol), testosterone (T), cortisol (F), KN and dithiothreitol (DTT) were purchased from Wako (Osaka, Japan). Ethinyl estradiol (EE2) was purchased from Sigma-Aldrich (St. Louis, USA). E2, E1, E3 and EE2 were first solubilized in ethanol to obtain 8 mM stock solutions, and were diluted with 50% ethanol. NAP-5 columns (Sephadex G-25 DNA grade) were purchased from G.E. Healthcare (Little Chalfont, UK). 3-Aminopropyltriethoxysilane (APTES) was obtained from Tokyo Chemical Industry (Tokyo, Japan). Slide glass and cover glass were purchased from Matsunami Glass Industry (Osaka, Japan).

The following buffers were used: (1) 6× binding and washing buffer without NaCl (6× BWB): 12 mM Tris–HCl (pH 7.5), 3.0 mM KCl, 1.2 mM MgCl_2_ and 0.6 mM CaCl_2_. (2) Tris–HCl, MgCl_2_ and NaCl buffer (TMN): 30 mM Tris–HCl (pH 7.5), 100 μM MgCl_2_ and 10 mM NaCl. (3) 2× reaction buffer: 40 mM Tris–HCl (pH 7.5), 2.0 M NaCl and 20 mM MgCl_2_.

### Preparation of dense DNA-modified AuNPs using thiol DNA

AuNPs modified with DNA at a high density were prepared using thiolated ssDNA (thiol DNA) as described previously with a slight modifications.^20^ Before the modification, the thiol DNAs were incubated with DTT and DTT subsequently removed with a NAP-5 column. AuNPs were sonicated for 1 minute and the purified DNA was added at 5 nmol mL^−1^ together with 0.01 M sodium phosphate (pH 8.0) and 0.01% Tween20. The DNA/AuNP solution was incubated at 50 °C for 20 min. Subsequently, the concentration of NaCl was increased to 0.05 M, the solution sonicated for 10 s and again incubated at 50 °C for 20 min. This process was repeated to increase the NaCl concentration stepwise to 0.2 M for the immobilization of thio18 followed by incubation at 50 °C for 24 h. For the immobilization of thio18 + T5 and thio18 − T5, the final NaCl concentrations before incubation at 50 °C for 24 h were adjusted to 0.4 and 0.1 M, respectively, to obtain the same amount of the immobilized DNA. For the immobilization of the low amount of thio18 (approximately 100 molecules per AuNP) for the comparison with DNA_adsorbed_–AuNPs, the DNA/AuNP solution was incubated in 0.1 M sodium phosphate buffer, pH 8.0 and 0.01% Tween20 at 40 °C for 12 h. Excess DNA was removed by replacing the supernatant after centrifugation with 0.01% Tween20. The washing process was repeated three times and finally the thiol DNA-modified AuNPs (DNA_brush_-AuNPs) were re-dispersed in MQ water at a concentration of 650 pM. The amount of modified DNA was estimated as reported previously.^8^ Briefly, the prepared DNA_brush_-AuNPs was incubated with 1% 2-ME at room temperature for 16 h to remove the immobilized DNA. Subsequently, the solution was centrifuged (25 °C, 8000 rpm, 15 min), and the amount of immobilized DNA in the supernatant was estimated using the QuantiFluor ssDNA System (Promega, Madison, USA). The amount of thiol DNAs immobilized on AuNPs was estimated to be approximately 1000 molecules per AuNP otherwise indicated.

### Inhibition of unspecific AuNPs aggregation by dense thiol DNA modification

4 μL of DNA_brush_-AuNPs were mixed with 4 μL MQ water and 2 μL 6× BWB and incubated at room temperature for 30 min. 2 μL of KN (various final concentrations ranging from 0 to 150 μM) were added and incubated at room temperature for 30 min. Following incubation, 8 μL of 50 mM NaCl were added to the sample and incubated for 60 minutes. Pictures were taken with a digital camera and UV-VIS spectra (390–900 nm) were recorded with a TECAN Safire^2^ microplate reader (Männedorf, Switzerland). The OD_750_/OD_530_ ratio was calculated in order to quantify the degree of AuNP aggregation. For comparison, AuNPs modified with ssDNA by physical adsorption (DNA_adsorbed_-AuNPs), which was conventionally used, were prepared as described.^27^ Briefly, the unmodified AuNPs were sonicated for 1 min and centrifuged (8000 rpm, 15 min, 4 °C). The supernatant was removed, and the AuNPs samples were re-dispersed in MQ water at a concentration of 700 pM. 4 μL of this 700 pM AuNPs stock were mixed with 4 μL of 500 nM non-thiolated DNA and 2 μL of 6× BWB at room temperature for 30 min allowing non-specific DNA adsorption. The amount of adsorbed ssDNA was estimated to be approximately 100 molecules per AuNP by measuring the amount of DNA in the supernatant after incubation with 2-ME as described.^27^

### E2 detection using aptamer-hybridized DNA-modified AuNPs

4 μL of prepared DNA_brush_-AuNPs were mixed with 4 μL of 200 nM E2 aptamer and 2 μL of 2× reaction buffer, and incubated at room temperature for 30 min to prepare aptamer-hybridized DNA-modified AuNPs (Apt-DNA_brush_-AuNPs). 2 μL of E2 diluted in 50% ethanol from the stock solution to yield indicated final concentrations (0, 0.1, 0.5, 1.0, 5.0, 10, 20 μM) were added to the Apt-DNA_brush_-AuNPs solution and mixed at room temperature for 30 min. Following incubation, 8 μL of 2× reaction buffer were added and incubated for 60 min to induce AuNP aggregation. Pictures were taken with the digital camera and UV-VIS spectra (390–900 nm) were measured using a microplate reader. The degree of nanoparticle aggregation was evaluated by calculating the OD_750_/OD_530_ ratio. The detection limit (LOD) was determined as a concentration that was higher than the 3*σ* line,^30^ where *σ* denotes the standard deviation of zero-concentration background data.

### TEM transmission electron microscopy (TEM)

Transmission electron microscopy (TEM) was performed in order to confirm the aggregation of the AuNPs. 5 μL sample was spotted on collodion-film coated TEM copper grids and allowed to adsorb. Distilled water was added and excess suspension was removed from the grid using filter paper. The grids were air-dried prior to analysis. Samples were examined with an excitation voltage of 80 kV using a JEM-1400Plus TEM (JEOL, Tokyo, Japan).

### E2 detection in the presence of KN

Apt-DNA_brush_-AuNPs were applied to detect E2 in the presence of KN. 2 μL of KN (300 μM, f. 30 μM) solution including E2 at different final concentrations (0, 0.1, 0.5, 1, 5, 10, 20 μM) were added to 10 μL of prepared Apt-DNA_brush_-AuNPs solution, and incubated at room temperature for 30 min. Then 8 μL 2× reaction buffer were added to induce AuNPs aggregation and incubated for 60 min. For comparison, AuNPs modified with E2 aptamer by physical adsorption (Apt_adsorbed_-AuNPs) were prepared as follows. Briefly, 4 μL of 700 pM AuNPs were mixed with 4 μL of 250 nM E2 aptamer solution and 2 μL of TMN buffer at room temperature and incubated for 60 min to allow for non-specific adsorption of the aptamer DNA on the AuNP surface. 2 μL of KN (300 μM, f. 30 μM) solution including E2 at different final concentrations (0, 0.1, 0.5, 1, 5, 10, 20 μM) were added to 10 μL Apt_adsorbed_-AuNP solution, and incubated at room temperature for 30 min. Following incubation, 8 μL of 62.5 mM NaCl were added and incubated for 60 minutes to induce AuNP aggregation. Pictures of the solutions were taken and the UV-VIS spectra (390–900 nm) were measured. Subsequently, the OD_750_/OD_530_ ratio was calculated to quantify the degree of AuNP aggregation.

A sample of environmental water was collected from an irrigation canal on the campus of Ehime University. The water was filtered and spiked with E2 (0, 0.1, 0.5, 1, 5, 10, 20 μM) and 30 μM KN. These synthetic solutions were used to test the detection system and procedures were carried out as described above.

### Detection of estrogenic compounds with the presented system

Estradiol (E2), estrone (E1), estriol (E3), ethinyl estradiol (EE2), deoxycholic acid (DCA), cholesterol (Chol), testosterone (T), cortisol (F) at 0, 5 and 20 μM were used as examples of estrogenic compounds and the response of the AuNP system was assessed.

From a previous report the environmentally relevant concentration ratios of E1, E2 and E3 were obtained and mixed model estrogen samples were prepared.^23^ For the mix of E1 + E2, the concentrations were 0 + 0 μM, 1.7 + 3.3 μM, 6.7 + 13.3 μM, respectively. For E1 + E2 + E3, the concentrations were 0 + 0 + 0 μM, 2.1 + 1.7 + 1.2 μM, and 8.3 + 6.7 + 5.0 μM, respectively. These mixed model samples were used to test the detection system using Apt-DNA_brush_-AuNPs with the procedures described above.

### Detection of E2 using a smartphone dark field microscope

A prototype of a low-cost smartphone dark-field microscope was built. The mobile phone is a Huawei P30, the oil dark field condenser (U-DCW) and the 10× lens (CACHN10xIPC/0.25) were both from Olympus (Tokyo, Japan). The light source is an Akku-Arbeitsstrahler (FL1400R) by ANSMANN (Assamstadt, Germany) and the adapters were custom-made by the machine workshops at ETH Zürich and Ehime University. For comparison, DFM images using a conventional benchtop DFM were obtained using a BX53 microscope (Olympus) equipped with a UP73 CCD camera, UPlanFLN 60× objective lens, and U-DCW dark field condenser as described.^8^ Apt-DNA_brush_-AuNPs solutions incubated with different concentrations of E2 (0, 0.1, 0.5, 1, 5 and 10 μM) were prepared as described above. A AuNP sample (3 μL) was dropped on a slide glass treated with APTES and covered with a cover glass as described.^27^ Images acquired with a conventional DFM, as well as images obtained with the smartphone DFM, were subjected to an digital color analysis process as reported previously.^27,31^ In brief, the DFM images were split into red, green and blue channels and the intensity of each component image was obtained to calculate the RGB component ratio using ImageJ software.

## Results and discussion

### Inhibition of non-specific AuNP aggregation by dense thiol DNA modification

Previously, a number of compounds non-specifically interacting with AuNPs and thereby causing their aggregation have been reported.^18,19^ Antibiotics, compounds frequently occurring in environmental samples, have been described to cause non-specific aggregation.^19^ In this study we report the dense and strong loading of thiolated DNA (DNA_brush_) on AuNPs (Fig. 1a) to inhibit non-specific interaction between nanoparticles and contaminating compounds. The antibiotic KN was chosen as a model of a contaminating compound inducing non-specific AuNP aggregation. KN is an antibiotic and this class of substances is assumed to potentially occur in river water samples, which will be analyzed with the sensor system described below. We hypothesized that if the formation of non-specific aggregates by KN can be suppressed, this kind of surface modification could potentially suppress non-specific aggregation caused by other compounds.

The effect of the inhibition of aggregation based on the difference in the DNA immobilization method on the AuNP surface (Fig. 1a) was compared. The results of adding KN to these nanoparticle solutions at different concentrations are shown in Fig. 1c. Aggregation occurs at 50 nM KN and higher when the AuNPs were functionalized with DNA by physical adsorption (DNA_adsorbed_-AuNPs). In comparison, when thiol DNA was immobilized at a high density (DNA_brush_-AuNPs), non-specific aggregation induced by KN was significantly suppressed.

In order to quantitatively confirm the result of the visual inspection in Fig. 1c, the UV-Vis spectra were recorded. To determine the degree of aggregation, the OD_750_/OD_530_ ratio as an aggregation parameter was calculated. Fig. 1d shows an increase of the aggregation parameter depending on the KN concentration in non-thiol DNA-modified AuNPs. Aggregation parameters remained unchanged in thiol DNA modified AuNPs even at high KN concentration (30 μM). In conclusion, non-specific aggregation caused by KN can be suppressed by coating AuNPs with thiolated DNA at high densities.

Next, effect of DNA length on the effect for prevention of non-specific aggregation using DNA_brush_-AuNPs, on which the same number of thiol DNAs were immobilized, was investigated (Fig. 1e). Interestingly, DNA_brush_-AuNPs using longer DNA showed more stability against KN, while DNA_brush_-AuNPs with shorter DNA was less stable, suggesting that the amount of negative charge has significant effect on the prevention effect of non-specific aggregation.

In order to further elucidate the inhibition mechanism, we examined the effect of immobilization method by comparing DNA_brush_ and adsorbed DNA where the same amount of DNA was modified on AuNPs (Fig. 1f). Interestingly, AuNPs with DNA_brush_ was more stable against KN than adsorbed DNA-AuNPs, suggesting that immobilization method is important for reduced nonspecific aggregation.

It was also shown that non-specific aggregation of AuNPs by tetracycline (TET) was suppressed by a DNA brush, whereas TET induced non-specific aggregation for DNA_adsorbed_-AuNPs (Fig. S1, ESI†). Wang *et al.* reported that positive charge and hydrophilicity are potential characteristics of compounds relevant for the induction of non-specific AuNP aggregation.^19^ It is noted that change in the zeta potential of DNA_brush_-AuNPs by the addition of KN was negligible in the buffer solution, while binding of KN was observed in a buffer without salt (ESI, Fig. S2†). Importantly, addition of 20 mM NaCl induced release of KN. These results suggest that although KN could potentially bind to DNA_brush_-AuNPs by electrostatic interaction, the binding of KN to DNA_brush_-AuNPs is prevented in the presence of salt.

It has been suggested that AuNPs with a high density of DNA layer have excellent colloidal stability by both electrostatic and steric stabilization from the DNA.^32^ Fujita *et al.* showed that the stability of DNA_brush_-AuNP against salt could be related with thermal fluctuation of DNA on the surface.^33^ Thus, it is plausible that steric hindrance by DNA_brush_ could prevent non-specific aggregation by KN. This is consistent with the result above showing DNA_brush_ is more effective for the prevention effect of non-specific aggregation the than the same amount of adsorbed DNA.

### Estradiol detection using aptamer-hybridized DNA-modified AuNPs

**Fig. 2:**
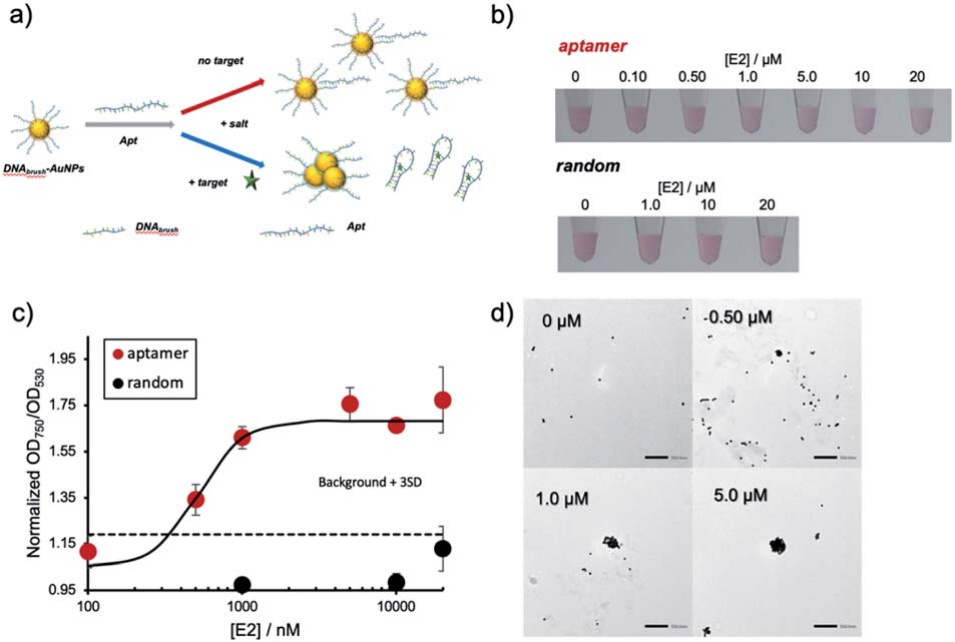
Detection of E2 using aptamer-hybridized DNA-modified AuNPs (Apt-DNA_brush_-AuNPs). (a) Schematic depicting the detection mechanism with Apt-DNA_brush_-AuNPs. (b) Analysis of the color of AuNP solutions when subjected to increasing concentrations of E2. Comparison of the response of the system to E2 when scrambled DNA (random) was hybridized instead of E2 aptamer. (c) Normalized OD_750_/OD_530_ of the solutions shown in (b). The averaged values of three different samples tubes were shown. (d) TEM images of AuNPs showing aggregation at increasing concentrations of E2 (scale bar = 500 nm).

In the next step, the created DNA_brush_-AuNPs were modified with an aptamer (Apt-DNA_brush_-AuNPs) to introduce a sensor function. As a proof-of-concept study, E2 was used as a model target, commonly occurring water pollutant causing endocrine disruption in humans and wildlife. Fig. 2a illustrates the detection scheme using an E2-aptamer immobilized by hybridization to the immobilized thiol DNA on the AuNP surface. This mechanism exploits the detaching of the aptamer from the complementary DNA to form aptamer-E2 complexes, a feature already used in the production of the aptamer.^29^ Thus we utilized the same aptamer sequence including the overlap sequence for hybridization, and hypothesized that if this aptamer is hybridized to the DNA immobilized on AuNPs, it will dissociate from the DNA upon addition of E2. Due to the negative charge of the DNA, the aptamer modification increases the dispersion stability of AuNPs and enables dispersion at higher salt concentrations. Once the target molecule is present, the aptamers detach from the AuNPs and aggregation is induced.

Fig. 2b shows the result of the visual inspection of the response of Apt-DNA_brush_-AuNPs to E2 and indicates a change of the color of the solution depending on the E2 concentration. The observed color changes arising from aggregation were quantified by UV-VIS, and the calculated OD_750_/OD_530_ ratios were shown in Fig. 2c. The values of the OD ratios increased depending on the E2 concentration. The control sample, scrambled DNA-hybridized DNA_brush_-AuNPs, did not show an E2 concentration-dependent color change and the OD ratio remained unchanged (Fig. 2b and c). These results demonstrate an aptamer sequence-specific E2-triggered AuNP aggregation. As another control experiment, when dithiothreitol (DTT) was added to Apt-DNA_brush_-AuNPs instead of E2, AuNP aggregation was observed (Fig. S3, ESI†). This could be due to detachment of DNA_brush_ by DTT. The limit of detection (LOD) of E2 of the presented detection system was evaluated to be 500 nM, at which concentration the OD ratio was higher than the 3*σ* line, where *σ* denotes the standard deviation of zero-concentration background data (Fig. 2c). This result was successfully reproduced (Fig. S4, ESI†). This is consistent with the dissociation constant of the aptamer (μM level) estimated from the previous report.^29^ The aggregation of the AuNPs caused by increasing concentrations of E2 was also confirmed by transmission electron microscopy (TEM) (Fig. 2d). The prepared DNA_brush_-AuNPs samples were stable at 4 °C at least 2 weeks, since the Apt-DNA_brush_-AuNP samples prepared after 2 weeks showed approximately 80% of E2 detection ability compared with the fresh sample (Fig. S5, ESI†).

### Detection of estradiol in the presence of kanamycin

**Fig. 3:**
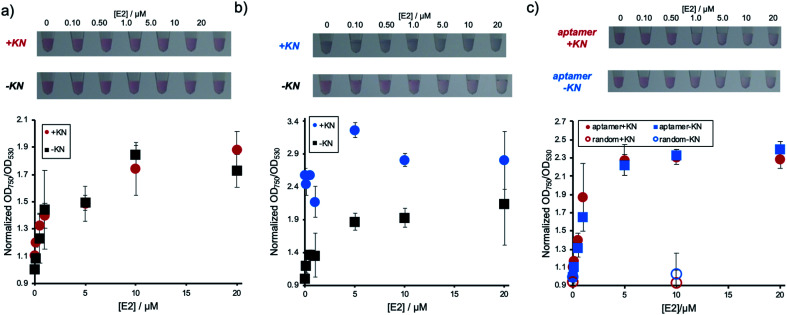
Detection of E2 with DNA_brush_-Apt-AuNPs in the presence of KN. The left panels show the color of the AuNP solution and the right panels show the normalized OD_750_/OD_530_. OD ratios of the blank sample were defined as 1. (a) E2 detection using DNA_brush_-Apt-AuNPs (system presented in this work) in the presence of KN. (b) E2 detection using Apt_adsorbed_-AuNPs (conventional system, DNA physically adsorbed on AuNP surface) in the presence of KN. (c) E2 detection in a model environmental water sample using DNA_brush_-Apt-AuNPs in the presence of KN. The negative controls are AuNPs using scrambled DNA (random) instead of an aptamer specific for E2. The averaged values of three different samples tubes were shown.

Next, it was investigated whether the detection system could be used for a sample containing E2 and compounds, such as KN, which induce non-specific AuNP aggregation. The performance of the conventional detection system, where DNA has only been physically adsorbed, was experimentally compared to the immobilization method described above. Fig. 3a shows the result of E2 concentration dependent AuNP aggregation of the E2 aptamer detection system using Apt-DNA_brush_-AuNPs in the presence of KN. As shown in the figure, the changes in color and OD ratio depending on E2 concentrations were identical to the samples without KN. In contrast, when Apt_adsorbed_-AuNPs were used, in which the DNA aptamer has only been physically adsorbed on the AuNP surface, presence of KN induced AuNPs aggregation and E2 cannot be detected by color change (Fig. 3b). From these results, it was concluded that the target can be detected even in the presence of aggregation-inducing compounds by hybridizing the aptamer to AuNPs, on which DNA has been immobilized at a high density. It is also noted that detection of 500 nM E2 in the presence of KN was confirmed in another experiment (Fig. S4, ESI†).

These results hint towards a potential applicability of the presented detection system for the detection of a given analyte in a sample containing compounds inducing non-specific aggregation. Such samples could for example be of biological nature, such as serum and urine, or environmental samples such as sewage and river water, which all might contain E2. We then present a proof of concept for the E2 detection from environmental water. A synthetic environmental water sample was prepared by filtering water from an irrigation channel on the campus of Ehime University and spiking it with E2 at different concentrations. The water samples were added to aptamer-modified AuNPs through covalently immobilized DNA. The results are presented in Fig. 3c. It was confirmed that the solution color changed and the values of OD ratio increased depending on the E2 concentrations regardless of the presence or absence of KN. In conclusion, this detection method can be applied for the detection of a target even in an environmental water sample containing compounds known to induce non-specific AuNP aggregation. Since the current LOD (∼500 nM) was much larger than possible E2 concentrations in environmental water (pM–nM range in river water and ∼sub μM in slurries),^23^ pre-treatment of the samples such as enrichment by vaporization would be necessary for the actual application. It is noted that considering that a possible actual concentration of antibiotics in environmental water is nM range in the river water,^19^ DNA_brush_-AuNPs could be stable even in the presence of simultaneously enriched antibiotics.

### Evaluation of the specificity of the detection system

**Fig. 4:**
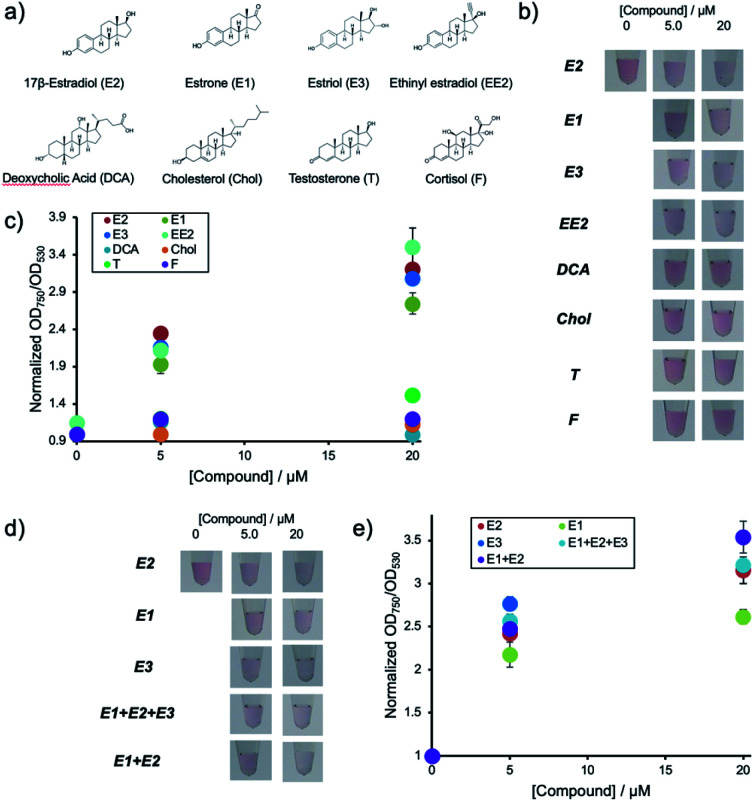
Evaluation of the specificity of the detection system using Apt-DNA_brush_ AuNPs. (a) Chemical structures of the compounds used to test the specificity of the detection system. (b) Visual inspection of the color of the AuNP solutions with different concentrations of the compounds added. (c) Normalized OD_750_/OD_530_ of the detection system caused by different compounds. (d) Colors of the AuNP solutions with each natural estrogen or their mixtures. (e) Normalized OD_750_/OD_530_ ratios to characterize the response of the detection system to natural estrogenic compounds and their mixes. The averaged values of three different samples tubes were shown.

Environmental water samples suspected of pollution with hormones usually contain mixtures of different natural estrogenic compounds. Thus the current system was subjected to different samples containing E1, E3, EE2, DCA, Chol, T and F. The chemical structures of the compounds are shown in Fig. 4a.

Fig. 4b shows the solution colors and Fig. 4c shows changes in OD ratio values for each of the compounds at different concentrations after their addition to aptamer-hybridized DNA-modified AuNPs (Apt-DNA_brush_-AuNPs). These results indicate that this detection system responds to E2 as well as E1 and E3. In particular, the signal of E3 is almost identical to the response caused by E2. This lies within expectations, as the chemical structure of E3 is very similar to E2 differing only by a hydroxyl group. Low signals for the other compounds even at concentrations up to 20 μM were observed. This indicates that the used aptamer shows estrogen-specific cross reactivity.

The detection system presented in this study could detect E2 and other estrogens such as E1 and E3. As environmental water samples usually contain mixtures of E1, E2 and E3 in different ratios the performance of the detection system in the analysis of samples containing mixtures was assessed. Two types of mixed estrogen samples with different concentration ratios based on the abundance ratios of E1, E2 and E3 in a Portuguese river were prepared as an example. Fig. 4d shows the solution color and Fig. 4e shows the OD ratio for each compound after addition of the mixtures to Apt-DNA_brush_-AuNPs. The OD ratio values increased depending on the compounds concentrations. Although a signal difference of about 20% between only E1 and only E2 is observed, the OD ratios in the estrogen mixture show the same increase rate as the OD ratio of E2. This result suggests that this detection system can be applied for the detection of the total amount of estrogens present in a sample. In any case, the cross-reactivity of the detection system depends largely on the recognition ability of used aptamer. Thus it may be possible to detect E2 more specifically or quantify a total estrogen amount more accurately by using an aptamer with different selectivity adequate to a use of detection.

### Detection of estradiol using a smartphone dark field microscope

**Fig. 5:**
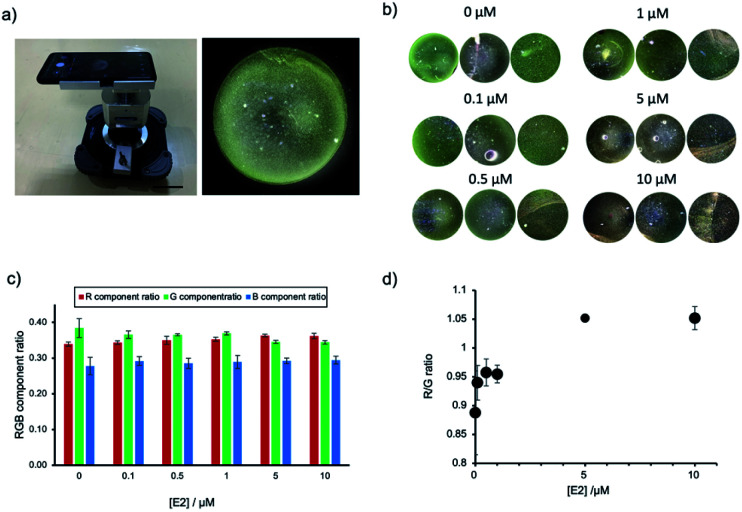
Characterization of E2-induced AuNPs aggregation by a smartphone DFM. (a) Photograph showing the smartphone setup. Scale bar = 5 cm. Example photo showing an AuNP solution visualized with the smartphone DFM. (b) Visualization of AuNPs aggregation caused by different concentrations of E2 (0, 0.1, 0.5, 1, 5 and 10 μM). For each concentration a smartphone DFM image was obtained. In each picture, images from three different experiments were subjected to RGB component ratio analysis. (c) Results of the RGB component ratio analysis of the images presented in (b). (d) R/G ratio obtained from the data in (c).

The application of AuNP-based sensors is currently restricted to a laboratory environment and requires trained users and specialized equipment. Guided by the setup of commercially available microscopes and the sketches of a low-cost smartphone DFM previously reported,^28^ we have designed and built our own prototype smartphone DFM (Fig. 5a and S6, ESI†). This smartphone DFM could observe scattering light from AuNP (Fig. 5a), and it was used to distinguish different degrees of AuNPs aggregation caused by increasing concentrations of E2 (0, 0.1, 0.5, 1, 5 and 10 μM). Fig. 5b shows the areas within the obtained smartphone microscope images, which were used for calculation of the RGB component ratios presented in Fig. 5c. The R/G component ratio analysis of the smartphone-obtained images confirmed an increase of the component of R with a decrease of the G component in an E2 dose-dependent manner (Fig. 5d). The aggregation was confirmed with a conventional benchtop DFM (Fig. S7, ESI†), supporting that this prototype of a smartphone DFM can be successfully used for the evaluation of AuNP aggregation.

## Conclusions

In this work we have suggested a method to prevent non-specific aggregation of AuNPs by KN, a potential co-contaminant of environmental water samples. Non-specific aggregation was prevented by introducing a dense DNA modification on the AuNP surface by using thiol DNA and forming Au–S bonds. Subsequently, in order to create a sensor moiety, an aptamer specific for E2 was hybridized to the DNA immobilized on the AuNP surface as a proof-of-concept study. The performance of this system in the detection of E2 was investigated. We have also characterized its applicability for the detection in samples containing E2 and model compounds, such as KN, inducing non-specific AuNP aggregation. Subsequently, we have demonstrated that the aptamer-based detection system for E2 works not only in pure water but also in a model environmental water from an irrigation canal spiked with E2 and KN. As environmental water samples suspected of pollution with hormones usually contain mixtures of different natural estrogenic compounds, the specificity of the system was tested with the most common compounds.

We also present advances in using a smartphone DFM to replace expensive and bulky laboratory equipment for the sensor readout potentially enabling future analysis applications in remote and resource-limited settings. For the whole system to become applicable for native environmental samples, the specificity and sensitivity of the sensor need to be increased. This could be achieved by using different aptamers or with a new aptamer engineered to possess a higher specificity for estradiol. Future trials will revolve around the detection of hormones in environmental samples at very low concentrations and automated image analysis in a smartphone application. With this result we hope to contribute to the translation of AuNP-based analytics with smartphones in the low-cost detection of hormones in aqueous sample matrices from laboratories to the field.

## Author contributions

T. Z. conceived the project. T. Z., Y.Y.-O. and A. O. designed the experiments. Y.Y.-O. performed all the experiments. Y. M. carried out TEM observation. T. M., M. M. and A. O. contributed AuNP surface modification. N. L., K. Y., W. S. and T. A. contributed smartphone DFM observation. Y.Y.-O., N. L. and T. Z. wrote the initial manuscript draft, and all the authors discussed the results and contributed to the manuscript preparation.

## Conflicts of interest

There are no conflicts to declare.

## Supplementary Material

RA-011-D0RA05149G-s001
