# Supplementary material for: Molecular detection using aptamer-modified gold nanoparticles with an immobilized DNA brush for the prevention of non-specific aggregation
Source: RSC Adv. 2021 Mar 24;11(20):11984–91. doi: 10.1039/d0ra05149g (PMC8696536; doi:10.1039/d0ra05149g)
Supplement: RA-011-D0RA05149G-s001 [file RA-011-D0RA05149G-s001.pdf]

Electronic Supplementary Material (ESI) for RSC Advances.
This journal is © The Royal Society of Chemistry 2021

Supplementary Information

Molecular detection using aptamer-modified gold nanoparticles with an immobilized DNA brush for the prevention of nonspecific aggregation

Yuki Yano-Ozawa[a], Nadine Lobsiger[a,b], Yu Muto[a,c], Takahiro Mori[a], Ken Yoshimura[a], Yuki Yano[a], Wendelin Jan Stark[b], Mizuo Maeda[d], Tsuyoshi Asahi[a], Atsushi Ogawa[e] and Tamotsu Zako[*a]

[a]*Department of Chemistry and Biology, Graduate School of Science and Engineering, Ehime University, 2-5 Bunkyo, Matsuyama, Ehime 790-8577 Japan E-mail:zako.tamotsu.us@ehime-u.ac.jp*
[b]*Institute for Chemical and Bioengineering, ETH Zürich, Wolfgang-Pauli-Strasse 10, CH-8093 Zürich, Switzerland*
[c]*Tokyo Research Center, TOSOH Corporation, 2743-1 Hayakawa, Ayase, Kanagawa 252-1123, Japan*
[d]*Bioengineering Laboratory, RIKEN Cluster for Pioneering Research, 2-1 Hirosawa, Wako, Saitama 351-0198, Japan*
[e]*Proteo-Science Center, Ehime University, 3 Bunkyo, Matsuyama, Ehime 790-8577, Japan*

Table of Contents

Fig. S1 Prevention of non-specific aggregation by tetracycline using DNA-brush.

Fig. S2 Zeta potential of DNA_{brush}-AuNPs in the presence of KN

Fig. S3 Effect of DTT on Apt-DNA_{brush}-AuNPs

Fig. S4 Detection of 500 nM E2 with DNA_{brush}-Apt-AuNPs in the absence or presence of KN

Fig. S5 Storage of DNA_{brush}-AuNPs

Fig. S6 Set up of a smartphone dark field microscope

Fig. S7 Characterization of estradiol-induced nanoparticle aggregation by a conventional dark field microscope.

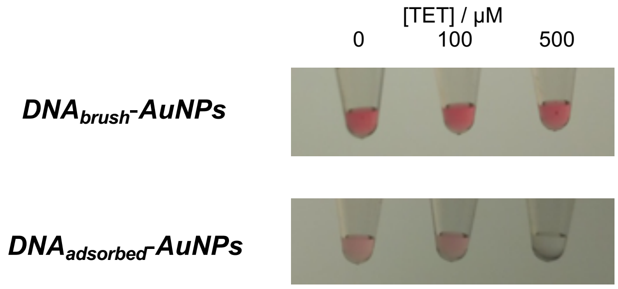

Fig. S1 Prevention of non-specific aggregation by tetracycline using DNA-brush. Tetracycline (TET) was added to DNA_{brush}-AuNPs and $DNA_{adsorbed}$-AuNPs solutions at the indicated concentrations.

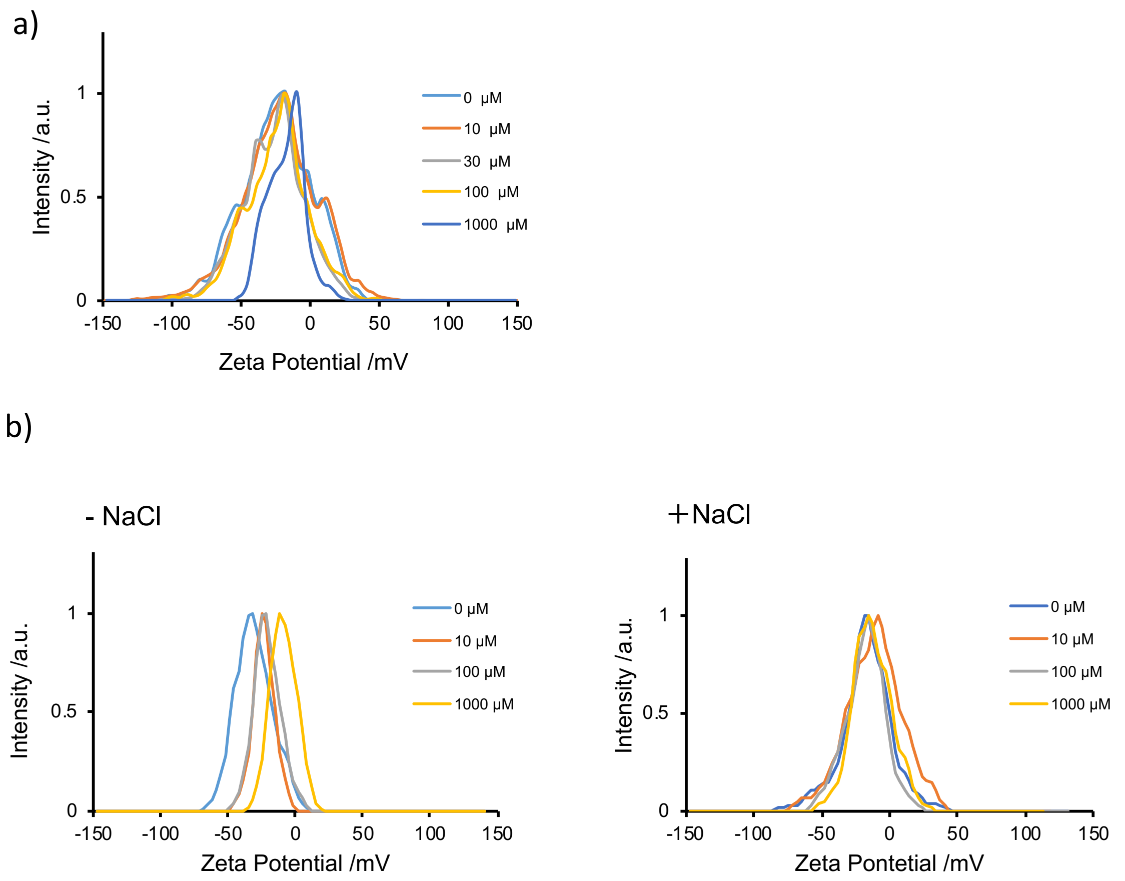

Fig. S2 Zeta potential of DNA_{brush}-AuNPs in the presence of KN. a) Zeta potential of DNA_{brush}-AuNPs in the presence of various concentrations of KN in TN buffer (20 mM Tris-HCl, pH 7.5 and 20 mM NaCl). Zeta-potential of 50 pM DNA_{brush}-AuNPs was measured using Zetasizer-Nano ZS (Malvern, Worcestershire, UK). b) Zeta potential of DNA_{brush}-AuNPs in the presence of various concentrations of KN in a Tris buffer (20 mM Tris-HCl, pH 7.5)(left). The solutions were taken, and 20 mM NaCl was added. Then the zeta potential values were measured (right).

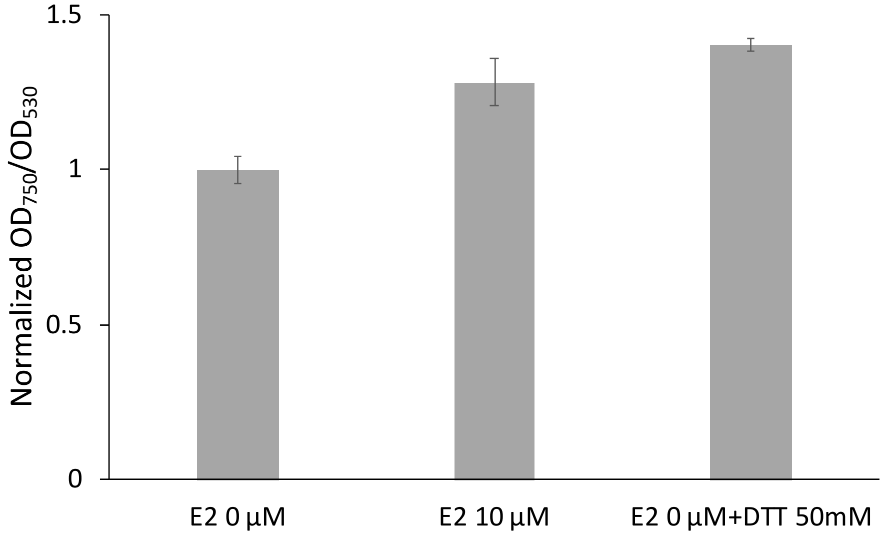

Fig. S3 Effect of DTT on Apt-DNA_{brush}-AuNPs. E2 (10 μM) or DTT (50 mM) was added to Apt-DNA_{brush}-AuNPs, and incubated at room temperature for 30 min. Then the reaction buffer was added and incubated for 60 min to induce AuNP aggregation. The degree of nanoparticle aggregation was evaluated by calculating the OD_{750}/ OD_{530} ratio. Normalized OD_{750}/ OD_{530} values are shown.

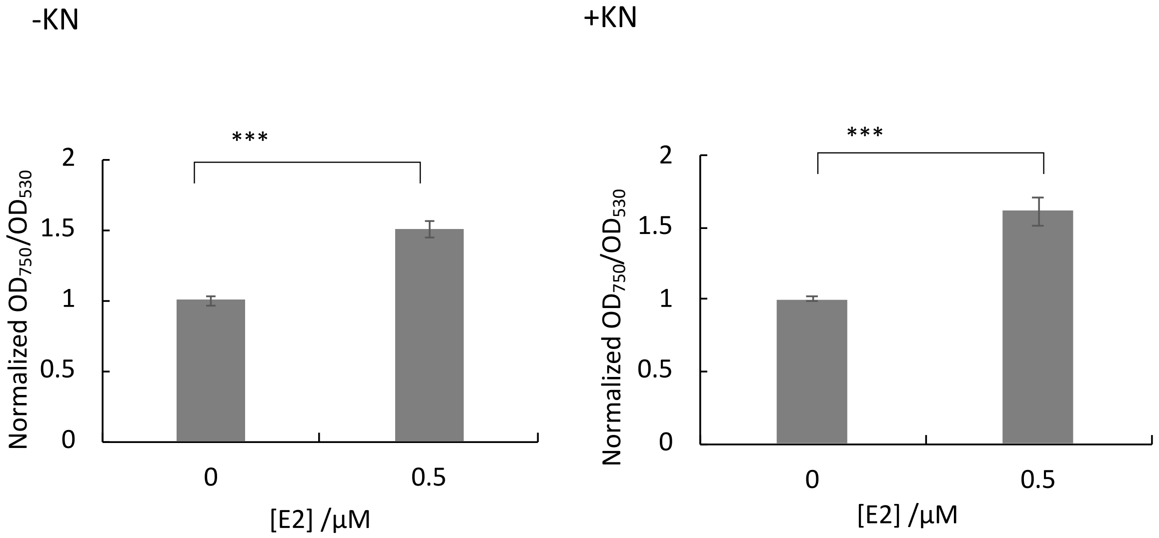

Fig. S4 Detection of 500 nM E2 with DNA$_{brush}$-Apt-AuNPs in the absence (left) or presence (right) of 30 µM KN was confirmed by another round of experiment. The normalized OD_{750}/OD_{530} values were shown. OD ratios of the sample without E2 were defined as 1. The averaged values of three different samples tubes were shown. ***, $p<0.005$

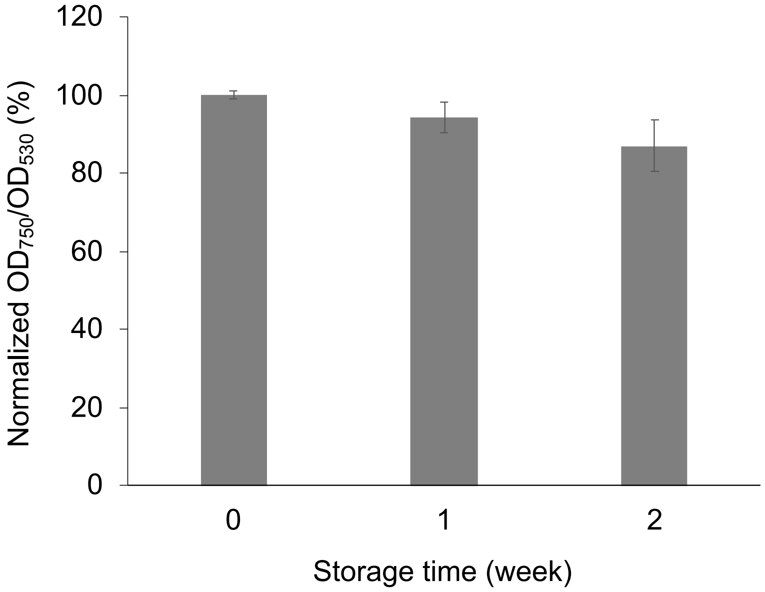

Fig. S5 Storage of DNA_{brush}-AuNPs. DNA_{brush}-AuNPs solution was stored at 4°C. After 1 or 2 weeks, Apt-DNA_{brush}-AuNPs were prepared for E2 detection. , E2 (10 μM) was added to Apt-DNA_{brush}-AuNPs, and incubated at room temperature for 30 min. After the addition of the reaction buffer, the samples were incubated for 60 min to induce AuNP aggregation. The degree of nanoparticle aggregation was evaluated by calculating the OD_{750}/ OD_{530} ratio. Normalized OD_{750}/ OD_{530} values are shown using the value of 0 week storage with a fresh DNA_{brush}-AuNPs sample as 100%.

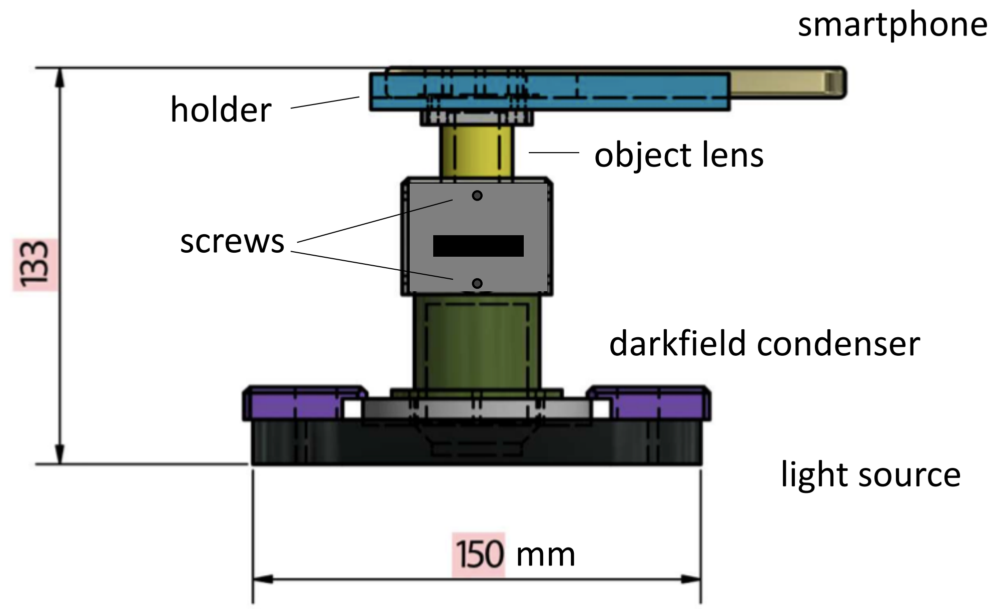

Fig. S6 Set up of a smartphone dark field microscope. The oil dark field condenser (U-DCW, Olympus) was attached to the light source (FL1400R, ANSMANN, Germany). The 10×object lens (CACHN10xIPC/0.25, Olympus) was attached with the smartphone (Huawei P30). The condenser and the lens were connected with the custom-made adapter with screws, by which the position was adjusted. The slide glass was inserted to the hole of the adapter. The cost of the total setup was approximately 1300 USD (condensor, 500; light source, 100; object lens, 200; smartphone, 500).

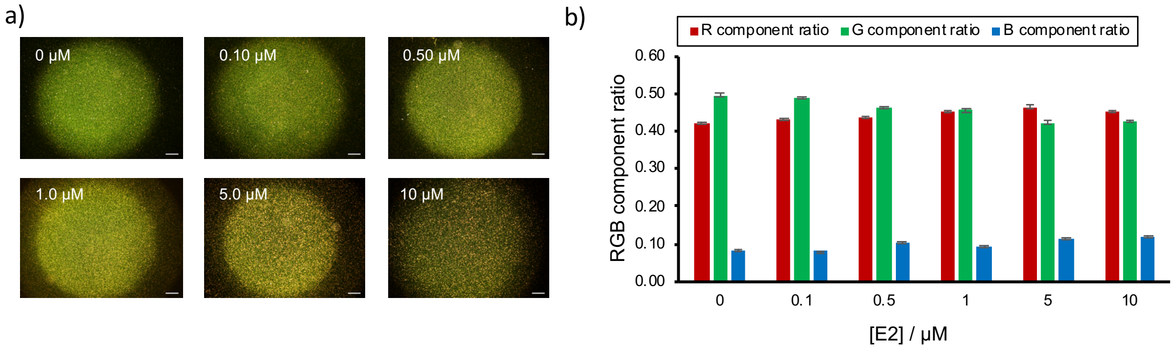

Fig. S7 Characterization of estradiol-induced nanoparticle aggregation by a conventional dark field microscope. a) Visualization of the analysis procedure of gold nanoparticle aggregation caused by different concentrations of estradiol (0, 0.1, 0.5, 1, 5 and 10 uM). For each concentration a smartphone microscope image was obtained. In each picture, three circular areas were subjected to RGB component ratio analysis. b) Results of the RGB component ratio analysis of the discs presented in a).
